# Critical Relaxation in the Quantum Yang–Lee Edge Singularity

**DOI:** 10.3390/e27020170

**Published:** 2025-02-06

**Authors:** Yue-Mei Sun, Xinyu Wang, Liang-Jun Zhai

**Affiliations:** 1 The School of Mathematics and Physics, Jiangsu University of Technology, Changzhou 213001, China; sunym@jsut.edu.cn (Y.-M.S.); wxy@jsut.edu.cn (X.W.); 2 The Jiangsu Key Laboratory of Clean Energy Storage and Conversion, Jiangsu University of Technology, Changzhou 213001, China

**Keywords:** critical relaxation, Yang–Lee edge singularity, parity-time symmetry breaking phase transition

## Abstract

We study the relaxation dynamics near the critical points of the Yang–Lee edge singularities (YLESs) in the quantum Ising chain in an imaginary longitudinal field with a polarized initial state. We find that scaling behaviors are manifested in the relaxation process after a non-universal transient time. We show that for the paramagnetic Hamiltonian, the magnetization oscillates periodically with the period being inversely proportional to the gap between the lowest energy level; for the ferromagnetic Hamiltonian, the magnetization decays to a saturated value; while for the critical Hamiltonian, the magnetization increases linearly. A scaling theory is developed to describe these scaling properties. In this theory, we show that for a small- and medium-sized system, the scaling behavior is described by the (0+1)-dimensional YLES.

## 1. Introduction

In 1952, Yang and Lee [1,2] established a way to understand phase transitions from the statistical mechanics by studying the partition function zeros, termed as Lee–Yang zeros, on the complex plane of the symmetry breaking field. Then, it was later pointed out that the edge of the Lee–Yang zeros is the branch point of the free energy [3] and the corresponding singular behavior, which is often referred to as the Yang–Lee edge singularity (YLES), can be described by the Landau–Ginzburg action with an imaginary cubic coupling [4]. So far, studies on the Lee–Yang zeros and the YLES have been extended to various systems, even beyond the statistical mechanics. Although the YLES is described by a non-Hermitian Hamiltonian and occurs in the complex parameter space, recent progress made in experiments has provided promising approaches to detect it [5,6,7,8,9,10,11,12,13,14]. In addition, the non-Hermitian Hamiltonian can be regarded as an effective model describing the dynamics in open quantum systems, which provide a powerful method for quantum state engineering and attract lots of theoretical, numerical and experimental studies [15,16,17,18,19,20,21,22,23,24,25,26,27,28,29,30,31,32,33,34,35,36,37,38,39,40,41,42,43,44]. Moreover, the YLES is shown to be a kind of dissipative phase transition associated with the parity-time (PT) symmetry breaking, which presents rich phenomena of quantum phases and has been observed experimentally [7,8,9,10,11]. Additionally, it has been shown that the scaling behavior of the spinodal decomposition belongs to the universality class of the YLES [45].

Currently, spurred by a series of recent experiments with cold-atom gases, understanding nonequilibrium behaviors has become one of the most challenging problems in quantum closed systems [46,47,48,49,50]. These studies include how statistical mechanics emerge under unitary time evolution, dynamical quantum phase transitions, universal scaling in the prethermal short time stage, and so on. On the other hand, it has recently been shown that in the driven dynamics of the YLES, the Kibble–Zurek mechanism, which predicts the emergence of the topological defects after crossing the point and has been confirmed in conventional quantum criticality, breaks down, although the full scaling theory is applicable [51,52]. Furthermore, the dynamics in the PT-symmetric system have been simulated in experiments, and power-law behaviors have been observed near the critical point [17]. This inspires the question as to how to characterize the relaxation dynamics.

To answer this question, we studied the relaxation critical dynamics of the YLESs in a quantum Ising model with the initial state being a completely polarized state. We show that for the critical Hamiltonian, which is exactly at its critical point, the magnetization linearly increases. For the paramagnetic Hamiltonian, the magnetization oscillates (quasi-)periodically with the period being inversely proportional to the gap between the lowest energy level, and for the ferromagnetic Hamiltonian, the magnetization decays to a saturated value. This phenomenon is in analogy with the slow dynamics in the classical critical system and the relaxation scaling of the prethermal dynamics in dynamical quantum phase transitions (DQPTs) [53,54]. A scaling theory was developed to understand these scaling behaviors. According to this scaling theory, we find that for small- and medium-sized systems, the relaxation dynamics is described by the (0+1)D critical exponents of the YLES. This scaling theory is confirmed by both the analytic and numerical results.

## 2. Model and Its Static Scaling

The Hamiltonian of the quantum Ising chain in an imaginary longitudinal field is given by [55]

(1) $$H=-\sum_{n=1}^{L}\sigma_{n}^{z}\sigma_{n+1}^{z}-\lambda \sum_{n=1}^{L}\sigma_{n}^{x}-ih\sum_{n=1}^{L}\sigma_{n}^{z},$$

where $\sigma_{n}^{x,z}$ are the Pauli matrices at site *n*, λ is the transverse field, and *h* is the longitudinal field which controls the distance to the critical point of the YLES. The YLES must occur for $\lambda >\lambda_{c}$ with $\lambda_{c}\equiv 1$ being the critical point of the conventional ferromagnetic–paramagnetic phase transition [56].

The order parameter for model (1) is defined as [55,57]

(2) M≡Re〈ΨgL|M^|ΨgR〉〈ΨgL|ΨgR〉,

in which $\hat{M}=\sum_{n}^{L}\sigma_{n}^{z}/L$, $\langle \psi_{g}^{L}|$ and $\psi_{g}^{R}\rangle$ are the normalized left and right eigenvectors satisfying $H|\psi_{g}^{R}\rangle =E_{g}|\psi_{g}^{R}\rangle$ and $\langle \psi_{g}^{L}|H=\langle \psi_{g}^{L}\left|E_{g}\right|$, and $E_{g}$ is eigenvalue with lowest real parts. We denote $h_{\mathrm{YL}}^{L}$ as the critical point of the YLES for fixed λ (>$\lambda_{c}$) and *L*. When $h<h_{\mathrm{YL}}^{L}$, M=0 and the energy spectra are real; in contrast, when $h>h_{\mathrm{YL}}^{L}$, M≠0 and the energy spectra become conjugate pairs. Exactly at $h_{\mathrm{YL}}^{L}$, the energy gap vanishes, in analogy to the case in the conventional quantum phase transition. Also, the equilibrium scaling behaviors of YLESs are described by the usual critical exponents. For instance, $\beta_{0}=1$, $\nu_{0}=-1$, $\delta_{0}=-2$, and the dynamic exponents $z_{0}=1$ [4,55,57]. However, there is a remarkable difference: the YLESs of model (1) can occur at both finite size and infinite size, while usual phase transitions can only occur at the thermodynamic limit. The YLES near $h_{\mathrm{YL}}^{L}$ belongs to the (0+1)D universality class.

## 3. Relaxation Scaling

Here, by comparing the relaxation scaling in classical criticality, we develop a scaling theory to describe the relaxation dynamics. Generally, the universal scaling behavior emerges after a transient time, $t_{M}$, in which the non-universal macroscopic dynamics dominated. For simplicity, let us set the initial state to be |Ψ(t=0)〉=|↓↓…↓〉, which is the steady state for h→∞. Since this state corresponds to an apparent fixed point of the scale transformation, the initial information should not be included in the scaling theory. In the following, we will denote $g_{L}$ as $g_{L}\equiv h-h_{\mathrm{YL}}^{L}$.

For small-sized and medium-sized systems, in the critical region of the (0+1)D YLES, after a microscopic transient time $t_{M}$, the relaxation behavior of *M* should satisfy

(3) $$M\left(t,g_{L}\right)=t^{-\frac{\beta_{0}}{\nu_{0}z_{0}}}f_{a}\left(g_{L}t^{\frac{\beta_{0}\delta_{0}}{\nu_{0}z_{0}}}\right),$$

in which $f_{a}$ is a scaling function. Although Equation (3) is similar to its counterpart in the classical relaxation, their scaling behaviors should be different. According to Equation (3), $M\propto t^{-\beta_{0}/\nu_{0}z_{0}}$ when $h=h_{\mathrm{YL}}^{L}$. This indicates that the absolute value of *M* increases linearly with time. In contrast, in the classical case, *M* decreases since ν is positive therein [58].

## 4. Relaxation for Single-Spin YLES

Here, we analytically solve the relaxation dynamics for L=1. In this case, there is no ferromagnetic coupling in model (1) and $h_{\mathrm{YL}}^{L=1}=\lambda$. After a transient time, $t_{M}$∼$\lambda^{-1}$, the dynamic process enters the universal stage. A time scale $t_{S}$, dictated by the energy gap $\Delta \propto g_{L}^{\nu_{0}z_{0}/\beta_{0}\delta_{0}}$, separates the universal evolution into two stages: the short time stage and the long time stage. In the short time region, in which $t<t_{S}$, *M* reads

(4) $$M\left(t,g_{L}\right)≃t\left[-\lambda +\frac{2\lambda^{2}}{3}g_{L}t^{2}+O\left({\left(g_{L}t^{2}\right)}^{2}\right)\right].$$

One can readily find that Equation (4) satisfies the (0+1)D scaling form of Equation (3). For $g_{L}=0$, the absolute value of *M* increases linearly; while for $g_{L}\neq 0$, the evolutions of *M* deviate from the linear increase towards different directions.

**Figure 1:**
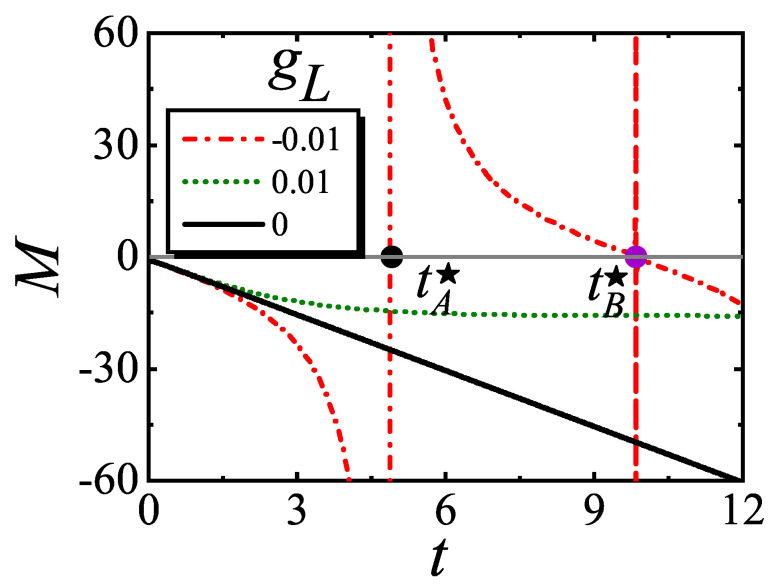
Critical relaxation of the YLES for L=1. Here, we use λ=5.

In the long time stage, $t\geq t_{S}$, for small $g_{L}$, *M* can be approximated by

(5) $$M\left(t,g_{L}\right)≃-\sqrt{\lambda /2}g_{L}^{-1/2}\tanh\left[\sqrt{2\lambda }\left(tg_{L}^{1/2}\right)\right],$$

except at some *critical instants* for $g_{L}<0$. One finds that Equation (5) also satisfies the scaling form of Equation (3) by deforming $f_{a}$ as $f_{a}\left(A\right)={\left(A\right)}^{1/\delta_{0}}f_{e}\left[{\left(A\right)}^{\nu_{0}z_{0}/\beta_{0}\delta_{0}}\right]$, where $f_{e}$ can also be regarded as a scaling equation. According to Equation (5), three cases are classified as follows: (i) for $g_{L}=0$, *M* still increases linearly; (ii) for $g_{L}>0$, *M* exponentially decays to its steady value $M≃-\sqrt{\lambda /2}g_{L}^{-1/2}$; (iii) for $g_{L}<0$, *M* oscillates with a period being inversely proportional to the energy gap Δ. We find that the former two cases are similar to the classical critical dynamics, while the last case is quite different from the classical case. The reason is that the dissipation dominates in the former two cases, in analogy to the classical criticality, while in the last case, the spectra are real and the dynamics are similar to the unitary evolution in quantum dynamics [59]. In Figure 1, the numerical results of relaxation dynamics with different $g_{L}$ for L=1 are plotted, verifying the analytically results.

For $g_{L}<0$, divergences arise at some critical instants $t^{★}$. We find that this phenomenon can be understood by the theory of the DQPT. In analogy to the fact that conventional phase transitions correspond to the Lee–Yang zeros of the partition function, the DQPT occurs at the time domain and is dictated by the zeros of the Loschmidt amplitude. Here, for the non-Hermitian system, the Loschmidt amplitude can be defined as [60,61]

(6) $$Z\left(t\right)=\langle \Psi {\left(0\right)}^{*}|e^{-2iHt}|\Psi \left(0\right)\rangle ,$$

which is just the denominator of the definition of *M*. (Here, the factor 2 in the evolution operator is just for convenience.) We find that the zeros of *Z* can be classified into two classes: (i) $t_{An}^{★}≃\left(\pi +2n\pi \right)/\left(2\sqrt{2\lambda }\Delta \right)$, with n∈Z. This class has been included in Equation (5). So the dynamics near $t_{An}^{★}$ are still described by Equation (3). (ii) $t_{Bn}^{★}≃\left(2\pi +2n\pi \right)/\left(2\sqrt{2\lambda }\Delta \right)$. This class is not included in Equation (5) and the dynamics near $t_{Bn}^{★}$ are non-universal. The reason is that for $g_{L}<0$, the energy spectra are real and the evolution operator can bring some non-universal initial information back to the dynamics, in analogy to the revival phenomena in unitary quantum dynamics [62].

## 5. Relaxation for Finite-Size YLES

In this section, we numerically solve the Schrödinger equation to model Equation (1) to study the relaxation dynamics in the critical region of (0+1)D YLES.

**Figure 2:**
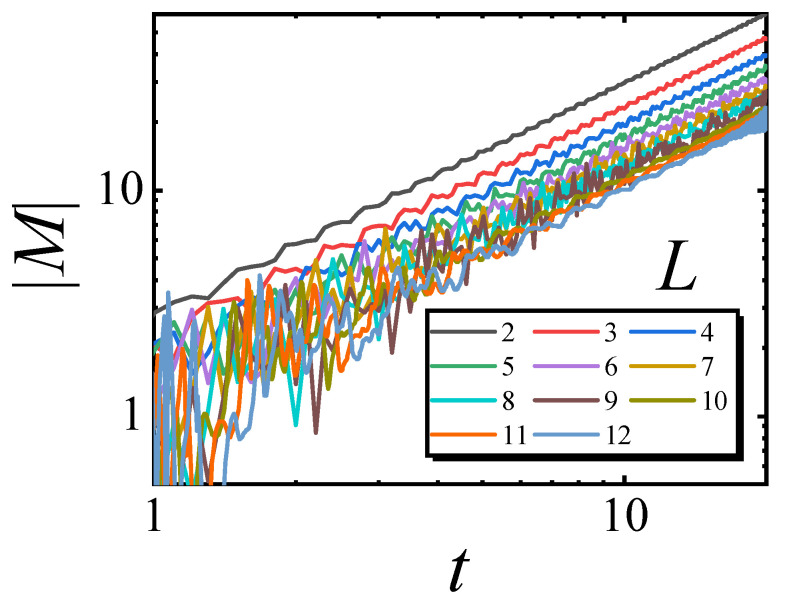
Critical relaxation with $g_{L}=0$ for different *L*. Here, we use λ=5, and the critical points of the YLES are $h_{\mathrm{YL}}^{L}=2.93334$, 2.56095, 2.43373, 2.37730, 2.34806, 2.33123, 2.32079, 2.31391, 2.30918, 2.30579 and 2.30331 for L=2,3,4,5,6,7,8,9,10,11 and 12, respectively. Double-logarithmic scales were used.

The critical relaxation with $g_{L}=0$ for small- and medium-sized systems was studied and is presented in Figure 2. It is shown that after a transient time, the curves of |M| versus *t* for different *L* are parallel lines in the log–log plot. The power-law fitting of the averaged slope for these curves is 1.003, confirming Equation (3).

For $g_{L}\neq 0$, the scaling function (Equation (3)) is rewritten as

(7) $$M\left(t,g_{L}\right)=g_{L}^{\frac{1}{\delta_{0}}}f_{a}\left(g_{L}^{\frac{\nu_{0}z_{0}}{\beta_{0}\delta_{0}}}t\right).$$

**Figure 3:**
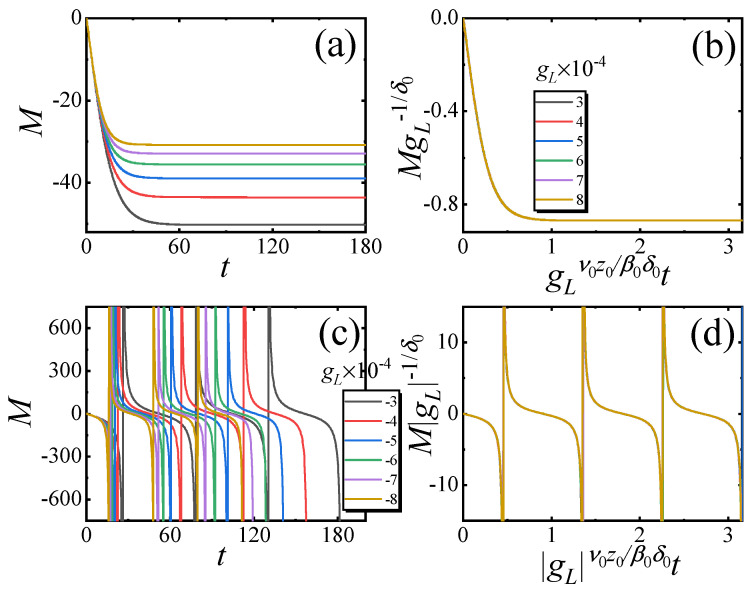
Critical relaxation for the small-sized system with nonzero $g_{L}$. (**a**) *M* versus *t* and (**b**) the rescaled curves according to Equation (7) with different $g_{L}>0$. (**c**) *M* versus *t* and (**d**) the rescaled curves according to Equation (7) with different $g_{L}<0$. Here, the lattice size is L=2, λ=5.

We first verify the scaling function (Equation (7)) for the small-sized system. In Figure 3a, *M* versus *t* with different $g_{L}>0$ for L=2 is plotted. We find that *M* also exponentially decays to its steady values. After rescaling *M* and *t* as $Mg_{L}^{-1/\delta_{0}}$ and $g_{L}^{\nu_{0}z_{0}/\beta_{0}\delta_{0}}t$, the rescaled curves match with each other, as shown in Figure 3b. In Figure 3c, *M* versus *t* with different $g_{L}<0$ for L=2 is plotted. It is shown that the evolution of *M* will exhibit periodic divergence. After rescaling *M* and *t* as $Mg_{L}^{-1/\delta_{0}}$ and $g_{L}t^{\beta_{0}/\nu_{0}z_{0}}$, the rescaled curves match each other, as shown in Figure 3d. These results confirm Equation (7).

Since the Loschmidt amplitude is the denominator term for *M*, the divergent behavior of *M* with $g_{L}<0$ also indicates the occurrence of DQPT. The period of the occurrence of DQPT *T* satisfies

(8) $$\begin{array}{c} T∼\Delta^{-1}. \end{array}$$

**Figure 4:**
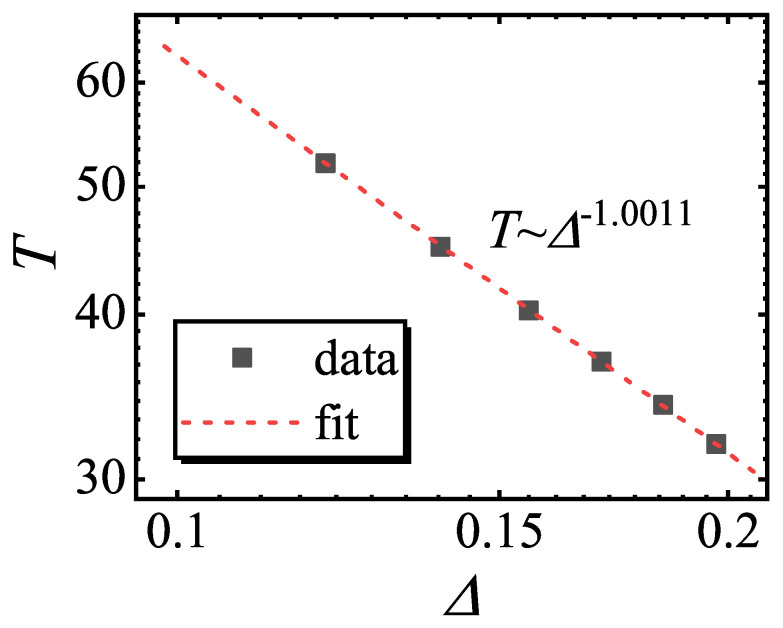
The period of the divergence of *M* in Figure 3c as a function of the energy gap between the lowest energy levels Δ. Double-logarithmic scales are used. Power-law fitting yields the slope −1.0011.

In Figure 4, the period of the divergence of *M* in Figure 3c versus Δ is plotted. A power-law fitting yields that *T*∼$\Delta^{-1.0011}$, confirming Equation (8). More importantly, we find that DQPT only occurs for the paramagnetic Hamiltonian, which may be because that a critical point is included in the quench process only in this case. This result also verifies the necessary conditions for the occurrence of DQPT [63].

**Figure 5:**
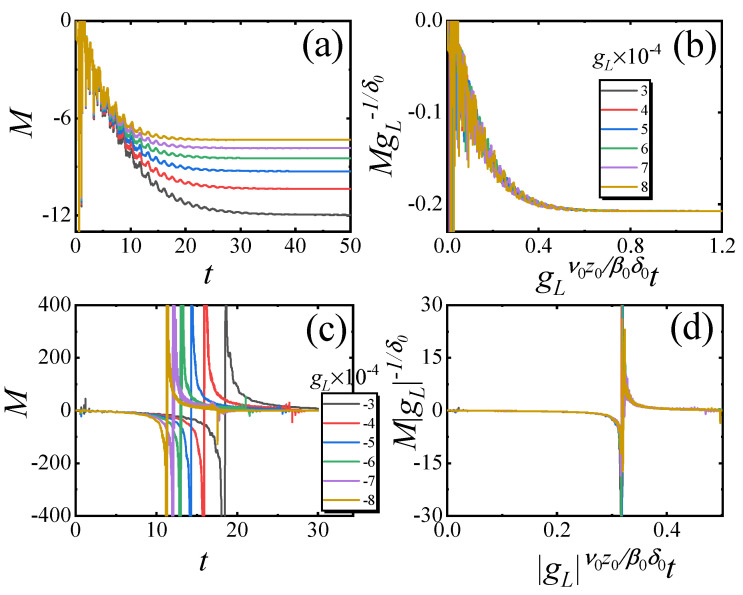
Critical relaxation for the medium-sized system with nonzero $g_{L}$. (**a**) *M* versus *t* and (**b**) the rescaled curves according to Equation (7) with different $g_{L}>0$. (**c**) *M* versus *t* and (**d**) the rescaled curves according to Equation (7) with different $g_{L}<0$. Here, the lattice size is L=12, λ=5.

For the medium-sized system, we choose the lattice size L=12. The numerical results for $g_{L}>0$ are plotted in Figure 5a. *M* exhibits exponential decay over time to stable values, similar to what is observed in small-sized systems; however, the curves become more rugged due to the increased involvement of excited states in the evolution process. After rescaling *M* and *t* according to Equation (7), the rescaled curves match with each other, as shown in Figure 5b. In Figure 5c, *M* versus *t* for $g_{L}<0$ is plotted. One finds that the divergent behavior still occurs periodically in the rugged curves. After rescaling, these rescaled curves collapse onto each other, as shown in Figure 5d. These results indicate that the (0+1)D scaling theory is still applicable in describing the relaxation dynamics of the medium-sized system.

## 6. Summary

In summary, we have studied the critical relaxation dynamics in the (0+1)D YLES critical region with the initial state being a completely polarized state. Through the analysis and numerical calculation, it was found that there are significant differences in the relaxation dynamics behavior for different types of Hamiltonian. For the critical Hamiltonian with $g_{L}=0$, the absolute value of *M* always increases linearly with *t*; *M* diverges periodically for the paramagnetic Hamiltonian with $g_{L}<0$ with the period being inversely proportional to the gap between the lowest energy level; for the ferromagnetic Hamiltonian with $g_{L}>0$, *M* exponentially decays to stable values. A scaling theory for the critical relaxation in the (0+1)D YLES critical region was developed and was numerically verified in both small- and medium-sized systems. Moreover, we found that the DQPT only appears for the paramagnetic Hamiltonian with $g_{L}<0$, because the critical point is included in the quench process. It is worth noting that we have chosen a completely polarized state as the initial state here. However, for other initial states, although their microscopic transient times in dynamics will be different, these dynamics can also be described by these scaling laws.

YLES was first discovered in the experiment with FeCl_2_ from experimental high-field magnetization data [7,8]. Subsequently, various schemes for realizing YLES and Lee–Yang zeros have been proposed [9,10,11,12,13]. Very recently, Gao et al. have also measured the critical properties of YLES in open systems [5], and the unconventional scaling laws for dynamics are demonstrated. Therefore, we expect that our study can be verified in these experiments. When the size *L* of Equation (1) is larger, the dynamics near the critical region of the (1+1)D YLES phase transition point can be described by the scaling theory of (1+1)D YLES. Moreover, it will also exhibit overlapping behavior with the critical region of the (0+1)D YLES. In addition, since recent experiments have explored the dynamical behavior of YLES at finite temperatures [5], it is also interesting to study the relaxation dynamics at finite temperatures. These are left for future works.

## Data Availability

The original contributions presented in this study are included in the article. Further inquiries can be directed to the corresponding author.

## Abbreviations

The following abbreviations are used in this manuscript:

YLES	Yang–Lee edge singularity
PT	parity-time
DQPTs	dynamical quantum phase transitions
